# Sn_x_P_y_ Monolayers: a New Type of Two-Dimensional Materials with High Stability, Carrier Mobility, and Magnetic Properties

**DOI:** 10.1186/s11671-020-03383-0

**Published:** 2020-07-29

**Authors:** Yan-Mei Dou, Chang-Wen Zhang, Ping Li, Pei-Ji Wang

**Affiliations:** School of Physics and Technology, University of Jinan, Jinan, 250022 Shandong People’s Republic of China

**Keywords:** Sn_x_P_y_, Monolayers, Carrier mobility, Magnetic properties, Optical property

## Abstract

Searching for two-dimensional (2D) group V materials with ferromagnetism, elastic anisotropy, and carrier mobility and tunable band structure is one key to developing constantly developing nanodevices. The 2D monolayers Sn_x_P_y_ with *x*/*y* (1/1, 1/2, 1/3, and so on) coordination number are studied based on the particle-swarm optimization technique combined with the density functional theory optimization. Its thermal stability can be confirmed by molecular dynamics at 70K and 300K, indicating that the novel 2D materials have a stable existence. The electronic band structures of four stable structures suggest that all the monolayers of Sn_x_P_y_ are fully adjustable and flexible tunable band gaps semiconductors under the biaxial strain. The monolayer of P$\overline{4}{2}_1$m-SnP_2_ with unique valence band structure can go from nonmagnetic to ferromagnetic by the hole doping because of the “Stoner criterion,” and Pmc2_1_-SnP_2_ is a direct-like gap semiconductor with in-plane elastic anisotropy to possess a high electron mobility as high as 800 cm^2^V^−1^ s^−1^ along the *k*_*b*_ direction, which is much higher than that of MoS_2_ (∼ 200 cm^2^V^−1^ s^−1^). The optical absorption peak of the material is in the ultraviolet region. These discoveries expand the potential applications of the emerging field of 2D Sn_x_P_y_ structures in nanoelectronics.

## Introduction

Two-dimensional (2D) binary compounds have attracted extensive attention in recent years due to their unique properties and can provide reliable guidance for its potential applications in nanoelectronics and optoelectronic devices [1]. For instance, graphene attracted great interest since its discovery due to properties and potential applications [2–6], whereas graphene has undetectable small band gap at room temperature, making it difficult to use in optoelectronic nanodevices. So, these difficulties promoted researchers to resolve to find 2D materials with an ideal band gap. In the following years, boron-nitride (BN) [7], MoS_2_ or other transition metal dichalcogenides [8–13], and transition-metal trichalcoge-nides [14, 15], among others are coming out. In recent years, graphene and other 2D materials of the group IV (silicone, stanene, and germanene [16]) have made good progress in scientific research. Except, 2D semiconductor materials belonging to the group V, especially phosphorene [17–19] and arsenene [20], are emerging as a new generation of contenders in the field of optoelectronic devices. Phosphene has broad application prospects in field-effect transistors, optoelectronic devices, spintronics, gas sensors and solar cells, and so on, while stanene, a 2D honeycomb-like structure, is considered to a new type of material with superior physical properties after graphene due to its strong electron spin-orbital coupling.

It is urgent to synthesize two kinds of elements to get multifunctional novel 2D materials. It is reported that alloying is often used to improve the properties of 2D materials to expand its applicability. For example, 2D MoS_2x_Se_2(1 − *x*)_ and WS_2x_Se_2(1 − *x*)_ nanosheets [21, 22] witness weird properties as tunable electronic, optical properties, and in-plane Negative Poisson’s Ratio with *x*/*y* (1/1, 1/2, 1/3, and so on) coordination number. For another example, 2D alloy material Si_x_C_y_ [23], B_x_C_y_ [24], and B_x_Si_y_ [25] show many novel characteristics (newfangled structure, electronic, and mechanical properties) by the first-principles calculation which are different from those of pure ground state. Because 2D phosphorene and stanene monolayers have novel properties, Sn and P elements were be compound with various stoichiometries.

In this work, we built few structures by the particle swarm optimization (PSO) algorithm. Then, we singled out the four most stable structures of 2D Sn_x_P_y_ monolayers with different coordination numbers and investigated the electronic properties on the basis of density functional theory (DFT) optimization. The calculated electronic band structures suggest that all the stable or metastable monolayers with different coordination numbers are semiconductors with an indirect band gap. More importantly, the Pmc2_1_-SnP_2_ monolayer is a direct-like gap semiconductor with a finite band gap of 0.92 eV in the infrared-light region. But beyond that, the Pmc2_1_-SnP_2_ structure is a direct-like gap semiconductor that possesses high electron mobility of ∼ 800 cm^2^V^−1^ s^−1^, which is much higher than that of MoS_2_ (∼ 200 cm^2^V^−1^ s^−1^). The monolayer of P$\overline{4}{2}_1$m-SnP_2_ structure with a unique valence band structure can go from nonmagnetic to ferromagnetic by the hole doping because of the “Stoner criterion.” The calculated electronic band structures suggest that all the monolayers of Sn_x_P_y_ are semiconductors with flexibly tunable band gaps under the biaxial strain, permitting strain engineering of four structures band gaps within nearly the whole visible-light range.

## Computational Methods

In order to guarantee a thorough search of the structural diversity, various *x* and *y* selecting from one to six are taken into account on the basis of the particle-swarm optimization (PSO) algorithm [26]. Results of the search delivered the monolayer structures are relatively steadily only for *y*/*x* ≧ 1.

To study the electronic structure of 2D Sn_x_P_y_ monolayers with different coordination number, our calculations were performed by using the plane-wave density functional theory (DFT) [27, 28] method to realize in the Vienna Ab-initio Simulation Package (VASP) [29–31]. Through the Generalized Gradient Approximation (GGA) to describe the exchange-correlation energy in the form of Per-dew–Burke–Ernzerhof (PBE) [32–35] and the electron-ion potential is described by the projection amplification wave method [33]. The cutoff energy of the plane-wave was chosen to be 500 eV energy for Sn_x_P_y_ systems, respectively. A sufficiently dense k point (9 × 9 × 1) of the reciprocal space was sampled in the Brillouin zone. The vacuum space perpendicular to the plane between neighboring super-cells is greater than 25 Å, eliminating the interaction between replications. In the two consecutive steps calculation, it is set as 10^5^ eV as the energy convergence value. During the geometric optimization, the atomic forces of all structures are less than 0.02 eV Å^−1^ by using the conjugate gradient method until the atoms reach their optimal position. In addition, we will use the supercell with 4 × 4 × 1 for ab initio molecular dynamic (AIMD) calculation when the Nosé algorithm [36] at 300K.

Carrier mobility is mainly affected by acoustic side wave scattering, optical side wave scattering, and ionized impurity scattering. Since the latter two are not as influential as the first one, the mobility we calculated includes the mobility under acoustic side wave scattering. Mobility mainly affects two performances of transistors: One is that the carrier concentration together determines the conductivity (the inverse of the resistivity) of the semiconductor material. Second, it affects the working frequency of the device. The main limitation of the frequency response characteristics of bipolar transistors is the time for minority carriers to cross the base region. Mobility is an important parameter to measure the conductivity of 2D semiconductor materials. It determines the conductivity of semiconductor materials and affects the working speed of devices. Thus, carrier mobility is controlled by phonon dispersion and can be described by the deformation potential (DP) theory which is proposed by Bardeen and Shockley [37]. So, the carrier mobility in 2D materials can be expressed as [38, 39]

$$ {\mu}_{2D}=\frac{2e{\mathrm{\hslash}}^3{C}^{2D}}{3{k}_BT{\left|{m}^{\ast}\right|}^2{E}_1^2} $$

where *e*, ℏ, and *k*_*B*_ are the electron charge reduced Planck and Boltzmann’s constant, respectively. And *T* is the temperature which set to 300K. Where m* is the effective mass, *E*_1_ is deformation potential constant, and *C*^*2D*^ is the in-plane stiffness.

The linear effect of the system on the light field under the small wave vector is determined by the imaginary part of the complex dielectric constant and the dielectric function which can be calculated by

$$ \upvarepsilon \left(\omega \right)={\varepsilon}_1\left(\omega \right)+i{\varepsilon}_2\left(\omega \right) $$

where the *ε*_1_(*ω*) and *ε*_2_(*ω*) are the real part of the function and the imaginary part, *ε*_1_(*ω*) can derive from the imaginary part *ε*_2_(*ω*) the dielectric function by Kramer–Kronig can be expressed [40]. The imaginary part of the dielectric function can be expressed as

$$ {\varepsilon}_2\left(\omega \right)=\frac{4{\pi}^2}{m^2-{\omega}^2}\sum \limits_{V,C}\underset{BZ}{\int }{d}^3k\frac{2}{2\pi }{\left|e\bullet {M}_{cv}\right|}^2\times \updelta \left[{E}_C\right.(k)-{E}_V(k)-\mathrm{\hslash}\left.\omega \right] $$

In addition, the absorption coefficient *I*(*ω*) was obtained by

$$ I\left(\omega \right)=\sqrt{2}\omega \left[\sqrt{\varepsilon_1^2\left(\omega \right)-{\varepsilon}_2^2\left(\omega \right)}-{\varepsilon}_1\left(\omega \right)\right]1/2 $$

where the *C* is the conduction band, the *V* is valence band states, *Ω* is the unit-cell volume, *m* is the mass of free electrons, *e* is the charge of free electrons, and *ω* is the frequency of incident photons.

## Results and Discussion

### Stability

First, four 2D Sn_x_P_y_ monolayers were considered to determine their energetic stabilities. Formation energy is an energy parameter in a thermodynamic system which is a key point to check the stability of the system. The relative stability of Sn_x_P_y_ monolayers can be confirmed by computing the formation energy and is calculated as

$$ {E}_{\mathrm{form}}=\left({E}_{\mathrm{total}}-{N}_{\mathrm{Sn}}{E}_{\mathrm{Sn}}-{N}_{\mathrm{P}}{E}_P\right)/\left({N}_{\mathrm{Sn}}+{N}_{\mathrm{p}}\right) $$

where *E* is the energy of a compound or a constituent element at a specific pressure. *N* is the number of atoms in the unit cell. The negative formation energy of the calculated system indicates that the configuration is stable or metastable [41]. The calculated formation energies of Sn_x_P_y_ monolayers are − 0.235, − 0.223, − 0.159, and − 0.016 eV/atom (shown in Table 1), respectively. According to its definition, a smaller value indicates higher stability. Obviously, P$\overline{6}$m2-SnP is the most stable of these four structures. More particularly, the high thermal stability of semiconductor materials is particularly important in the application of electronic devices. Here, the thermal stability of the Sn_x_P_y_ monolayers examined by using ab initio molecular dynamics (AIMD) simulations. Based on the symmetries of the space groups, we just calculate the stability of P$\overline{6}$m2-SnP for similar structures P$\overline{6}$m2-SnP and Pmc2_1_-SnP_2_ and Pmc2_1_-SnP_2_ for Pmc2_1_-SnP_2_and P$\overline{4}{2}_1$m-SnP_2_ structures. Results indicate that the average value of the total energy of the structure remains almost unchanged, and the structure remains unchanged after 1 ps, 3 ps, and 5 ps, suggest that Sn_x_P_y_ monolayers are thermally stable (in Figure S1). Then, we calculated phonon dispersion curves and have no imaginary vibrational frequencies implies that structures are dynamically stable (in Figure S1). Several methods have been reported in the literature to synthesize layered materials including micromechanical cleavage [2], epitaxial growth [42], chemical vapor deposition [43], and liquid exfoliation [44]. Some materials with the similar structure were successfully prepared experimentally. We have found some related reports that few layer GaSe nanosheets have been made into a high performance photodetector in the experiment [45]. In addition, the preparation, isolation, and rapid unambiguous characterization of large size ultrathin layers of MoS_2_, GaS, and GaSe deposited onto SiO_2_/Si substrates are reported [46].

**Table 1:** Lattice parameters and calculated formation energy of the Sn_x_P_y_ monolayers

Sn_**x**_P_**y**_ monolayers	Lattice parameters						Space group	Formation energy (eV/atom)
	*a*	*b*	*c*	*Alpha*	*Beta*	*Gamma*		
P$\overline{6}$m2-SnP	3.95	3.95	17.16	90	90	120	P$\overline{6}$m2	− 0.235
P$\overline{3}$m1-SnP	3.96	3.96	17.11	90	90	120	P$\overline{3}$m1	− 0.223
Pmc2_1_-Sn*P*_2_	3.71	10.78	15.83	90	90	90	Pmc2_1_	− 0.159
P$\overline{4}{2}_1$m-SnP_2_	7.59	7.59	14.66	90	90	126.69	P$\overline{4}{2}_1$m	− 0.016

**Fig. 1:**
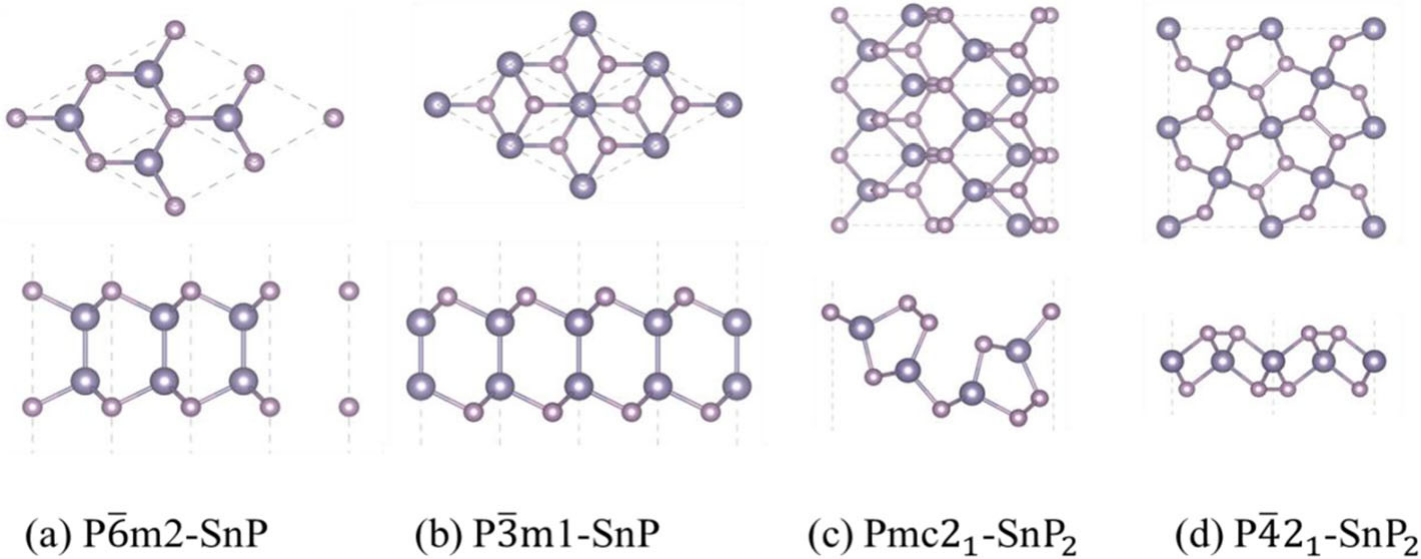
Top and side views of the atomic structures of Sn_x_P_y_ monolayers: **a** P$\overline{6}$m2-SnP, **b** P$\overline{3}$m1-SnP, **c** Pmc2_1_-SnP_2_, and **d** P$\overline{4}{2}_1$m-SnP_2_; the Fermi level (horizontal dashed line) is shifted to 0 eV. The heavy pink spheres represent Sn atoms, and light pink spheres represent P atoms

As plotted in Fig. 1a, b, the structures of P$\overline{3}$m1-SnP exhibit a structure similar to the P$\overline{6}$m2-SnP hexagonal phase. The Pmc2_1_-Sn*P*_2_ trigonal phase (Fig. 1c) exhibits that the *x*/*y* composition is further increased to 1/2. The material of a structure similar to P$\overline{4}$2_1_m-SnP_2_ was proved stable by theory calculation [47]. Also, a new study finds the structure of XY_2_ (Fig. 1d) is an indirect bandgap semiconductor, and it might susceptible to electric field and stress. We believe that the material we predict will have a successful preparation in the future with the development of technology.

### Electronic and Magnetic Properties

**Fig. 2:**
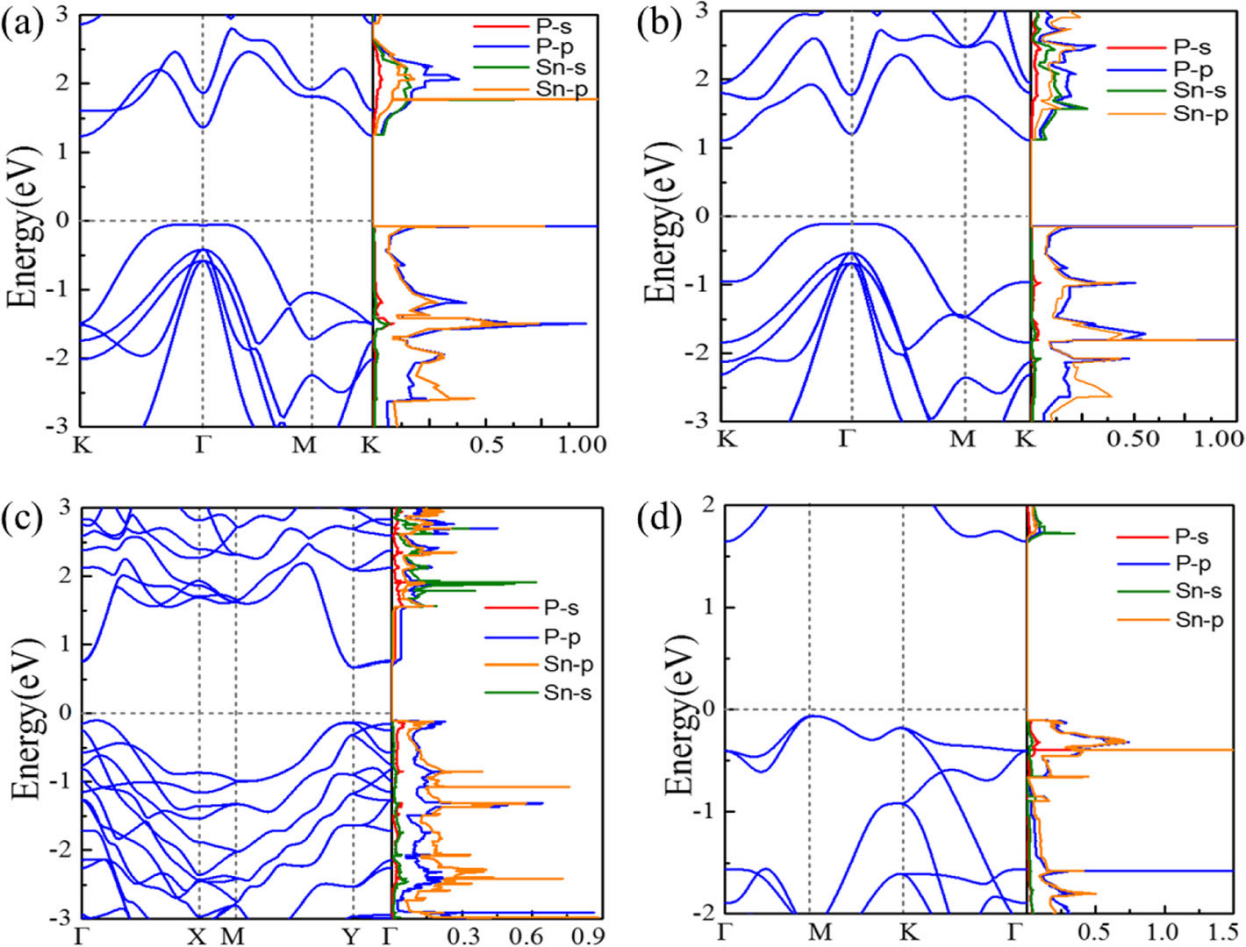
Computed electronic band structures and partial density of states of the VBM and CBM of Sn_x_P_y_ monolayers: **a** P$\overline{6}$m2-SnP, **b** P$\overline{3}$m1-SnP, **c** Pmc2_1_-SnP_2_, and **d** P$\overline{4}{2}_1$m-SnP_2_

The computed band structures and partial density of states of Sn_x_P_y_ monolayers are plotted in Fig. 2. As shown in Fig. 2a, the P6̅m2-SnP is an indirect semiconductor with a band gap of 1.19 eV. The valence band maximum (VBM) at Γ → K direction is contributed by the hybridized Sn-p and P-p orbitals, while the conduction band minimum (CBM) at K point derives from the hybridized Sn-s and P-p orbitals. P$\overline{3}$m1-SnP exhibits similar electronic band structures as the P$\overline{6}$m2-SnP counterpart but with a smaller band gap of 1.21 eV. The valence band dispersion of P$\overline{6}$m2-SnP and P$\overline{3}$m1-SnP near the Γ point and Fermi level (*E*_*F*_) is quite flat, given the rather high density of states (DOS) and a van Hove singularity around the VBM. The Pmc2_1_-SnP_2_exhibits direct-like gap semiconducting character (*E*_g_(direct) − *E*_g_(indirect) = 6 meV) with a band gap of 0.72 eV (see Fig. 2c). Its VBM is mostly attributed to P-p orbitals, while its CBM is mainly contributed by P-p orbitals and Sn-s orbital. The P$\overline{4}{2}_1$m -SnP_2_ is an indirect-gap semiconductor with an *E*_*g*_ of 1.79 eV, and the bands derive from is akin to Fig. 2c. More importantly, P$\overline{4}{2}_1$m-SnP_2_monolayer has a similar condition compare with Fig. 2a, b, a flat-band dispersion character around the VBM also arises, resulting in very high DOS and a van Hove singularity.

**Fig. 3:**
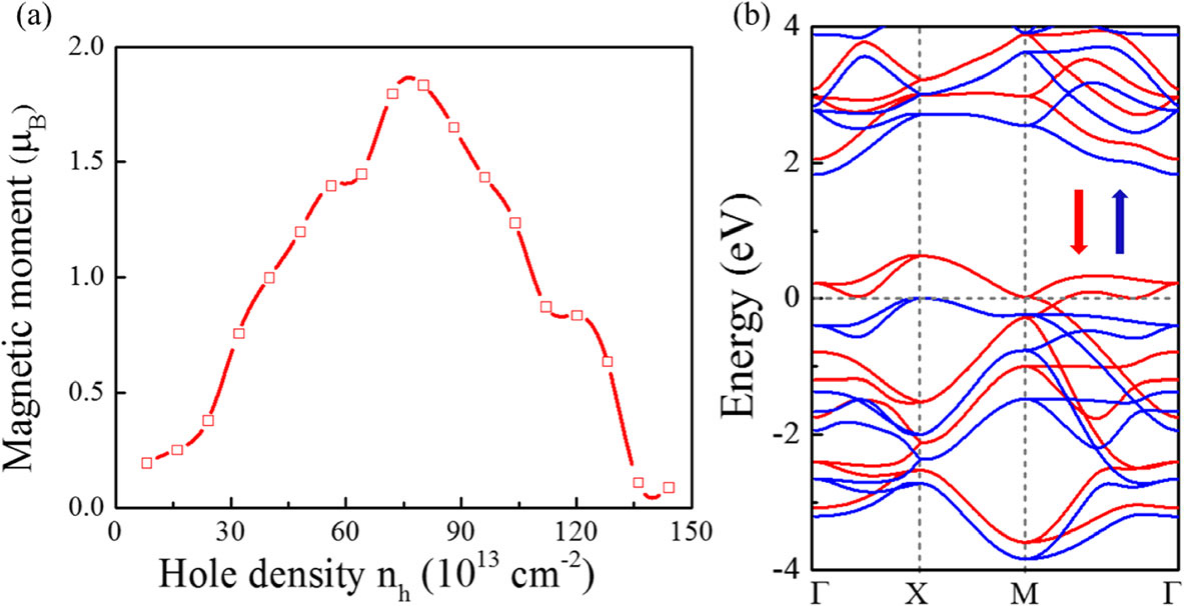
Ferromagnetism in P$\overline{4}{2}_1$m-SnP_2_ monolayer. (**a**) Spin moments vs hole density *n*_*h*_. **b** Computed valence band structure of P$\overline{4}{2}_1$m-SnP_2_ at *n*_*h*_ = 7.2 × 10^14^ cm^−2^. The spin-up and spin-down bands are shown in blue and red, respectively. The Fermi level is set to be 0 eV

According to Stoner criterion, spontaneous ferromagnetism occurs if the kinetic energy is smaller than the exchange splitting energy, which is if the DOS at *E*_*F*_ is high enough. Figure 2d shown the very high DOS around the VBM; the P$\overline{4}{2}_1$m-SnP_2_ may satisfy the Stoner criterion if its *E*_*F*_ is shifted to a position with high DOS through hole doping. As shown in Fig. 3a, the hole doping can introduce magnetic moments at appropriate doping concentrations. As expected, the computation result suggests that the P$\overline{4}{2}_1$m-SnP_2_ may be converted into a ferromagnetic ground state beyond the critical hole density. Among them, the hole density *n*_*h*_ can be expressed as *n*_*h*_ = *m*_*h*_/*S*_*cell*_, where *S*_*cell*_ and *m*_*h*_ are the area of the primitive cell and the number of holes introduced in the primitive cell. The injection of the hole into the monolayer P$\overline{4}{2}_1$m-SnP_2_ indeed leads to ferromagnetism. The magnetic moment exhibits a steep peak-like relationship with the hole density. Because an appreciable spin moment is induced by hole doping in the system, the energy band structure around the Fermi level has changed greatly due to spin splitting. Particularly, the spin-polarized band structure (shown in Fig. 3b) of P$\overline{4}{2}_1$m-SnP_2_ at 7.2 × 10^14^ cm^−2^ shows that the monolayer becomes a perfect half metal. So, we predict that the stable FM state with half metallicity can be realized in the P$\overline{4}{2}_1$m-SnP_2_ monolayer.

### Elastic Anisotropy and Carrier Mobility of Monolayer

**Fig. 4:**
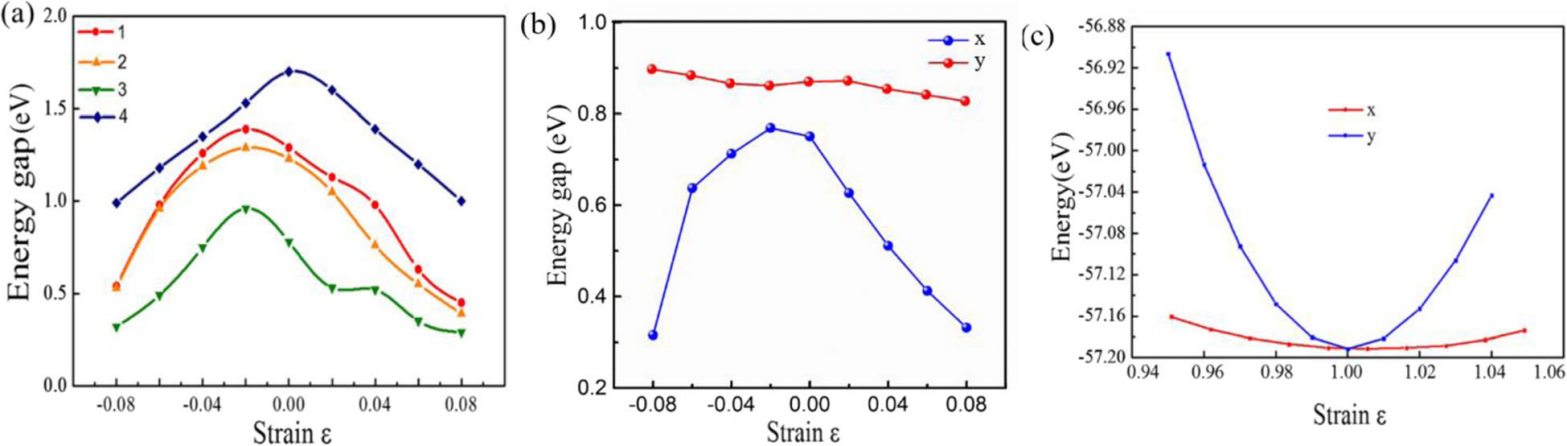
Strain-dependent electronic properties of structures: (1) P$\overline{6}$m2-SnP, (2) P$\overline{3}$m1-SnP, (3) Pmc2_1_-SnP_2_, and (4) P$\overline{4}{2}_1$m-SnP_2_. **b** Uniaxial strain dependent electronic properties of Pmc2_1_-SnP_2_. **c** Energy of the Pmc2_1_-SnP_2_ monolayer vs uniaxial stain

The strain effects on the electronic properties of the 2D monolayers Sn_*x*_P_*y*_ structures are also interesting. Figure 4a presents the energy gap variation under biaxial strain *ε*. The energy gap of Sn_x_P_y_ monolayers is markedly modulated according to some rules. For example, the energy gap of P$\overline{6}$m2-SnP decreases from 1.19 to 0.52 eV with the increasing tensile strain up to *ε* = 8%, first increasing from 1.12 to 1.36 eV for *ε* 2%, then decreasing from 1.36 to 0.51 eV. In addition, since the *a* and *b* of the lattice parameter of the Pmc2_1_-SnP_2_ structure are different, the changes of electronic properties are different along the *x*-axis and *y*-axis [48], as shown in Fig. 4b. It is obvious that when uniaxial strain is applied in different directions, the change in the *x* direction is different from the change in the *y* direction. Considering the range of energy gaps with the strain *ε*, the range of tunable band gap by in-plane strain almost covers the entire visible-light region based on the first-principles calculation.

Besides, we also investigated the effect of uniaxial stress, as shown in Fig. 4c. The in-plane stiffness *C*^*2D*^ (*C*^*2D*^ = [∂^2^*E*/∂*δ*^2^]/*S*_0_, where *S*_0_ is the area of 2D Pmc2_1_-SnP_2_ monolayer) can be obtained by fitting the parabola. Interestingly enough, the in-plane stiffness *C*^*2D*^ showing extremely obvious elastic anisotropy along *a* and *b* directions are calculated to be 12.1 and 105.6 N/m, respectively. Because the Pmc2_1_-SnP_2_ exhibits direct-like gap semiconducting character, the effective masses (*m*^∗^ = *ℏ*^2^(*∂*^2^*E*/*∂K*^2^)^−1^) of electrons (*m*_e_ is |m^*^_e_|) and holes (*m*_h_ is |m^*^_h_|) associated with the (quasi) direct semiconducting Pmc2_1_-SnP_2_ monolayer are also computed. The effective masses are listed (Table 2). Most interesting is the effective mass of electrons in the *k*_*b*_ direction (0.15 me) is much smaller than that in the *k*_*a*_ direction (1.31 me), indicating the easy drift of electrons in the *k*_*b*_ direction. There is another important parameter is DP constant *E*_1_ (*E*_1_ = d*E*_edge_/d*δ*) for electrons along *a* and *b* directions is calculated to be 5.36 and 11.57 eV, respectively. Surprisingly, the calculated carrier can be achieved ~ 800 cm^2^V^−1^ s^−1^ in the *k*_*b*_ direction. As a comparison, the carrier mobility of the MoS_2_ monolayer is ∼ 200 cm^2^V^−1^ s^−1^ in experiments [8]. However, the carrier mobility is just about ∼ 8 cm^2^V^−1^ s^−1^ in the *k*_*a*_ direction. Therefore, the high carrier mobility found in this study is of great significance for the study of electron transport.

**Table 2:** Effective masses of electrons and holes for Pmc2_1_-SnP_2_

	Pmc2_1_-SnP_2_	
|m^*^_e_|	1.31 (*K*_*a*_)	0.15 (*k*_*b*_)
|m^*^_h_|	0.42 (*k*_*a*_)	0.57 (*k*_*b*_)

### Optical Property

**Fig. 5:**
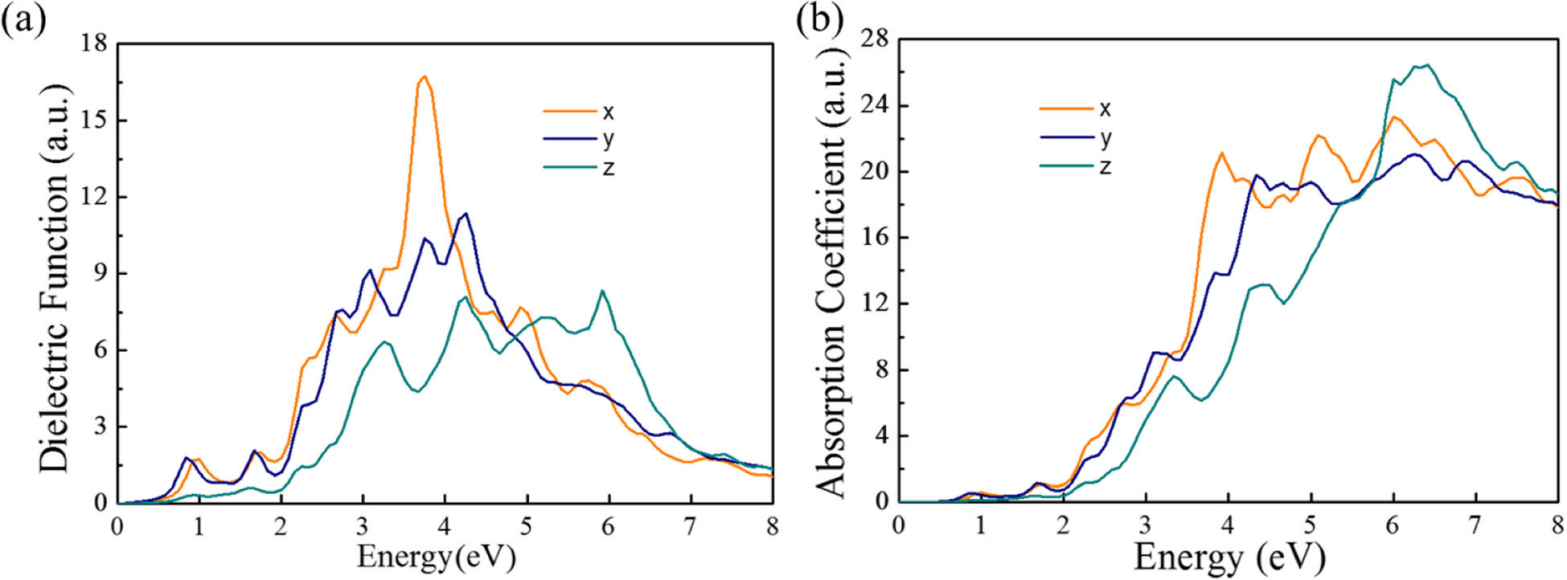
**a** Computed dielectric functions versus energy for Pmc2_1_-SnP_2_ along different incident light directions. **b** Computed imaginary absorption coefficient

The photoelectric properties of photoelectronic materials are characterized by dielectric function, photoconductivity, and absorption coefficient. The imaginary parts of the dielectric function are shown in Fig. 5a. Note that Pmc2_1_-SnP_2_ monolayer shows absorption starting at ∼ 0.70 eV, and there appear three main absorption peaks at ∼ 0.9, ~ 3.2, and ∼ 4.0 eV. As illustrated in Fig. 5b, it shows the absorbance in all three directions in the visible range and ultraviolet range for monolayer Pmc2_1_-SnP_2_. So, Pmc2_1_-SnP_2_ monolayer materials could be used for atomically thin solar-blind photodetectors for, e.g., efficient detection of flames.

## Conclusions

In conclusion, based on the PSO algorithms combined with first-principles calculations, we have identified several 2D Sn_x_P_y_ monolayers with the ratios of *x*/*y* = 1:1 and 1:2. Surprisingly, these novel monolayers also possess peculiar electronic and magnetic properties: the monolayer of P$\overline{4}{2}_1$m-SnP_2_ structure with unique valence band structure can go from nonmagnetic to ferromagnetic by the hole doping because of the “Stoner criterion”; the Pmc2_1_-SnP_2_ structure is a direct-like gap semiconductor with in-plane elastic anisotropy is found to possess high electron mobility as high as 800 cm^2^V^−1^ s^−1^ along the *k*_*b*_ direction, which is much higher than that of MoS_2_ (∼ 200 cm^2^V^−1^ s^−1^). The optical absorption peak of the material is in the ultraviolet region. These discoveries expand the potential applications of the emerging field of 2D Sn_x_P_y_ structures in nanoelectronics. These desirable properties of the multifunctional Sn_x_P_y_ monolayers provide promising great applications in electronics and optoelectronics.

## Supplementary information

**Additional file 1: Figure S1.** The total energy and kinetic energy versus the simulation steps; final equilibrium structures (inset) at T=300 K structures of Sn_x_P_y_ monolayers: (a) P$\overline{6}$m2-SnP, (b) Pmc2_1_-SnP_2_; the Fermi level (horizontal dashed line) is shifted to 0 eV).

## Data Availability

They are all in the main text and figures.
