# Supplementary material for: SnxPy Monolayers: a New Type of Two-Dimensional Materials with High Stability, Carrier Mobility, and Magnetic Properties
Source: Nanoscale Res Lett. 2020 Jul 29;15:155. doi: 10.1186/s11671-020-03383-0 (PMC7391462; doi:10.1186/s11671-020-03383-0)
Supplement: Supplementary file 1 — Additional file 1: Figure S1. The total energy and kinetic energy versus the simulation steps; final equilibrium structures (inset) at T=300 K structures of SnxPy monolayers: (a) P$\overline{6}$6¯m2-SnP, (b) Pmc21-SnP2; the Fermi level (horizontal dashed line) is shifted to 0 eV). [file 11671_2020_3383_MOESM1_ESM.docx]

$\mathbf{Sn}_{\mathbf{x}}\mathbf{P}_{\mathbf{y}}$ **Monolayers: A New Type of Two-dimensional Materials with High Stability, Carrier Mobility and Magnetic Properties**

Yan-Mei Dou, Chang-Wen Zhang, Ping Li, and Pei-Ji Wang*^^[[1]](#footnote-1)^^*

*School of Physics and Technology, University of Jinan, Jinan, Shandong, 250022, People’s Republic of China*


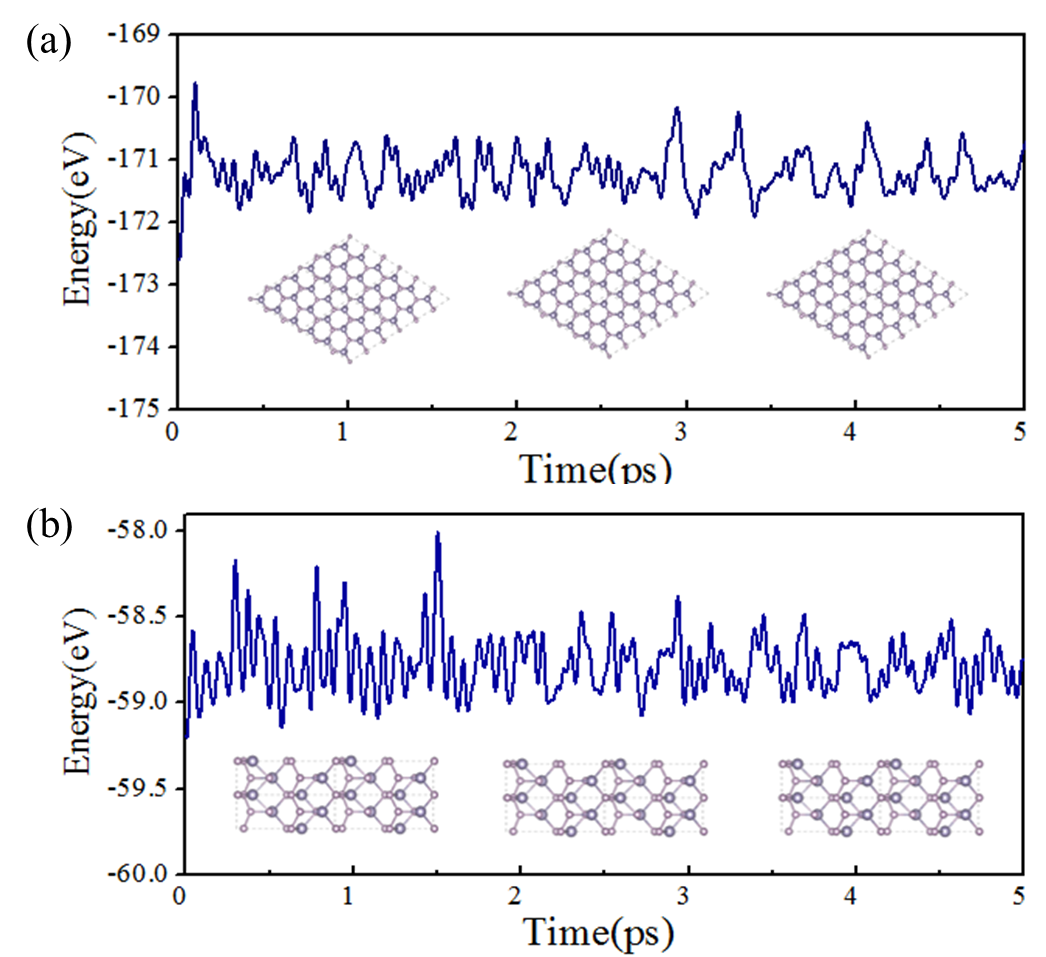


Figure S1. The total energy and kinetic energy versus the simulation steps; final equilibrium

structures (inset) at T=300 K structures of $\mathrm{Sn}_{x}P_{y}$ monolayers: (a) P$\bar{6}$m2-SnP, (b) Pmc$2_{1}$-Sn$P_{2}$; the Fermi level (horizontal dashed line) is shifted to 0 eV).


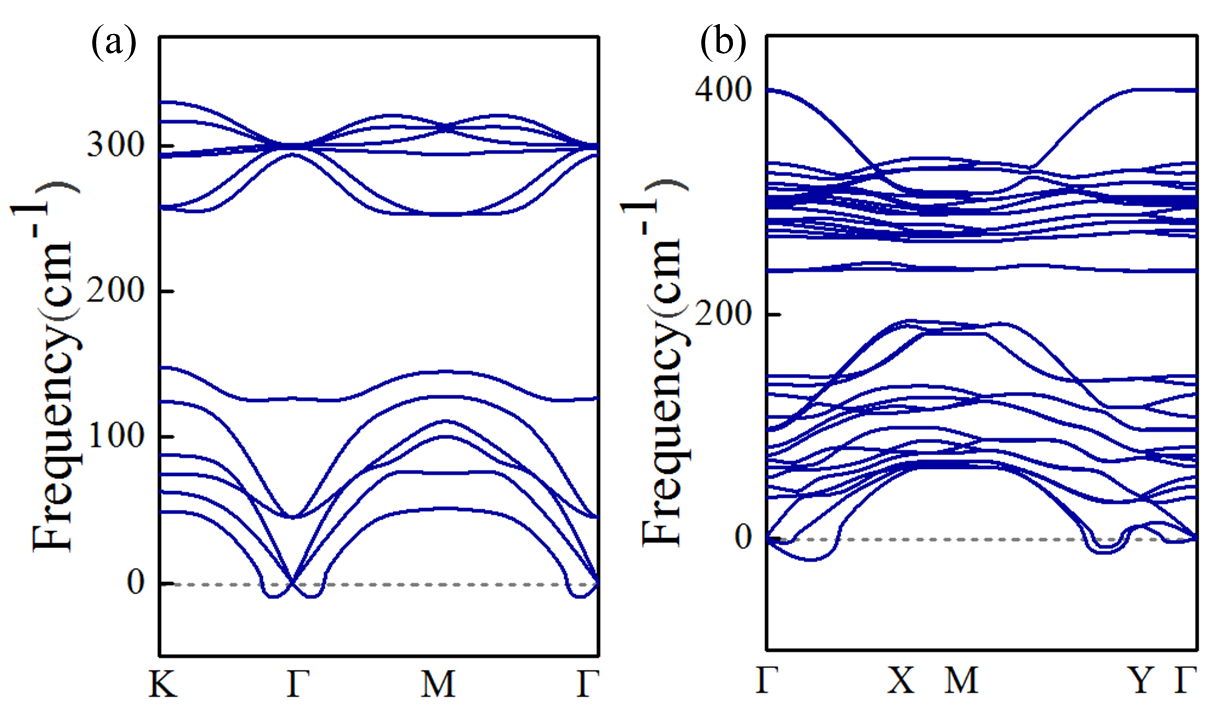


Figure S2. Computed phonon spectra of structures of $\mathrm{Sn}_{x}P_{y}$ monolayers: (a) P$\bar{6}$m2-SnP, (b) Pmc$2_{1}$-Sn$P_{2}$.

1. Corresponding author: E-mail: ss_wangpj@ujn.edu.cn [↑](#footnote-ref-1)
